# Applying motivational framework in medical education: a self-determination theory perspectives

**DOI:** 10.1080/10872981.2023.2178873

**Published:** 2023-02-22

**Authors:** Fraide A. Ganotice, Karen M. K. Chan, Siu Ling Chan, Sarah so Ching Chan, Kelvin Kai Hin Fan, May P. S. Lam, Rebecca Ka Wai Liu, Gloria H. Y. Wong, Grace Wai Yee Yuen, Jacqueline K. Yuen, Susanna Siu Sze Yeung, Ma Jenina N. Nalipay, Francis Hang Sang Tsoi, George L. Tipoe

**Affiliations:** a Bau Institute of Medical and Health Sciences Education, the University of Hong Kong, Hong Kong SAR, China; b Division of Speech and Hearing Sciences, the University of Hong Kong, Hong Kong SAR, China; c School of Nursing, the University of Hong Kong, Hong Kong SAR, China; d Department of Pharmacology and Pharmacy, the University of Hong Kong, Hong Kong SAR, China; e Department of Social Work and Social Administration, the University of Hong Kong, Hong Kong SAR, China; f Department of Medicine and Therapeutics, the Chinese University of Hong Kong, Hong Kong SAR, China; g Department of Psychology, the Education University of Hong Kong, Hong Kong SAR, China; h Department of Curriculum and Instruction, The Chinese University of Hong Kong, Hong Kong SAR, China

**Keywords:** self-determination theory, interprofessional education, scale application, construct validity, medical education

## Abstract

**Background:**

The application of self-determination theory in explaining student achievement has been well-established in various contexts. However, its application to medical education, particularly in interprofessional education (IPE) remains underexplored. Understanding how students’ motivation plays a role in students’ engagement and achievement is essential to optimize efforts to improve learning and instruction.

**Objective:**

This two-stage study aims to contextualize the SDT framework to IPE through the adaptation of the Basic Psychological Need Satisfaction to IPE (Study 1) and to demonstrate how SDT can be applied in IPE by examining a model of SDT constructs (Study 2) in predicting outcomes (behavioral engagement, team effectiveness, collective dedication, goal achievement).

**Design:**

In Study 1 (*n*=996), we adapted and validated BPNS-IPE using confirmatory factor analysis and multiple linear regression using data from 996 IPE students (Chinese Medicine, Medicine, Nursing, and Pharmacy). In Study 2 (*n*=271), we implemented an IPE program where we integrated SDT approaches and examined the relationship of SDT constructs with IPE outcomes using multiple linear regression.

**Results:**

Our data supported the three-factor structure (autonomy, competence, and relatedness) of BPNS-IPE, meeting the required model fit. Autonomy predicted team effectiveness (F=51.290, *p*<.05, R^2^=.580); competence predicted behavioral engagement (F=55.181, *p*<.05, R^2^=.598); while relatedness predicted significantly four IPE outcomes: behavioral engagement (F=55.181, *p*<.01, R^2^=.598), team effectiveness (F=51.290, *p*<.01, R^2^=.580), collective dedication (F=49.858, *p*<.01, R^2^=.573), goal achievement (F=68.713, *p*<.01, R^2^=.649).

**Conclusions:**

The SDT motivational framework can be adapted and applied in the IPE context to understand and enhance student motivation in medical education. Potential studies with the use of the scale are provided to guide researchers.

Literature indicates that patients can achieve more positive health outcomes when physicians deal with them in a more humanistic way. Hence, it is important not only to cultivate healthcare practitioners’ clinical skills but also to develop their ability to provide humanistically-oriented care [1]. One way to achieve this is through supporting their autonomy. Autonomy support is linked with the attainment of both cognitive (e.g., greater conceptual understanding) and non-cognitive (e.g., humanistic orientation towards patients and better psychological adjustment) learning outcomes [1]. Furthermore, autonomy-supportive instructors can facilitate not only a greater sense of autonomy and competence among healthcare students but also the value they place on the psychosocial aspects of healthcare [2]. Thus, the medical curriculum should emphasize the development of autonomous or intrinsic motivation in their programs to produce healthcare professionals who provide humanistically-oriented care.

Self-determination theory (SDT) is a human motivational theory that focuses on understanding autonomy functioning or sense of agency [3]. SDT posits that self-determined behaviors result in effective and lasting behavioral change and positive outcomes (e.g., better performance, adaptive functioning, and well-being). SDT has been studied at length in various domains, including education [4], sports [5], language learning [6], organizations [7], religion [8], health [9], virtual environment [10], and parenting [11], among others. SDT has also been introduced to medical education (e.g [2,12,13]). Surprisingly, limited attention has been given to the field of medical education, given how much it can contribute to developing competent and humanistic healthcare professionals in their interactions with patients.

Despite the strong empirical findings supporting the SDT assertions, its integration into medical education has been slow [14] and failed to draw traction in the context of healthcare interprofessional education (IPE) (see [15–17] for a few exceptions). The goal of interprofessional education (IPE) is to break down educational silos and help students develop their interprofessional competencies by allowing different but complementary social and medical specialties to learn from, about, and with one another to improve healthcare [18] and lessen medical errors [19]. A possible barrier to the integration of SDT into medical education, specifically in IPE, could be the lack of a valid and context-specific instrument to measure SDT constructs in this field. Although the bulk of SDT studies has been done in psychology and education, there are compelling theoretical reasons to support its applicability in medical education. For example, students, in general, need to have a strong sense of autonomy, competence, and relatedness to achieve learning outcomes [1].

The Basic Psychological Needs Satisfaction in General (BPNS-G) is designed to examine basic psychological needs satisfaction [20,21]. This scale has been largely used to examine general needs satisfaction [20,22–28]. It has been validated previously involving various populations: Americans [29], Englishmen [30], Indians [31], Germans, and Australians [32]. These studies provided support for the acceptability of BPNS-G and have been used in various contexts. The Basic Psychological Need Satisfaction and Frustration Scale is also available and has been adapted to various domains: Physical Education, Physical Exercise, Sports, Education, Romantic Relationships, Training, and Work Domain [33]. Despite this development, the scale has not been examined in IPE. Adapting the scale to IPE can help provide a much-needed motivational framework for explaining the learning outcomes in this field. This is especially true considering the observation that IPE studies appear atheoretical [34,35], which suggests the need to problematise IPE from theoretical lens and valid scales. The lack of utility of SDT in medical education could be attributed to the unavailability of a measure tailored to this context. Hence, we intended to adapt and validate BPNG-G to IPE. Developing a context-specific scale is an important first step to draw researchers’ attention in stimulating a discussion geared towards a conceptual understanding of the science and scholarship of IPE.

## Self-determination theory in interprofessional education

SDT specifies that intrinsic and extrinsic motivation are considered potent forces in shaping one’s behavior, with intrinsic motivation being the more autonomous, self-determined, and adaptive of the two [36]. This theory indicates that individuals are inherently growth-oriented following the satisfaction of universal psychological needs: needs for autonomy, competence, and relatedness. An important assertion of SDT is the notion of intrinsic motivation for which one’s behavior is induced by internal satisfaction.

IPE provides an ideal context to apply the theoretical assertions of SDT. In particular, IPE and SDT are complementary. Provisions for meeting the needs for autonomy, competence, and relatedness posited by SDT are in alignment with the aims inherent to IPE. In healthcare and social care, IPE and interprofessional collaborative practice (IPP) are important parts of curricula that can cultivate intrinsic motivation or the promotion of a sense of autonomy.

## The current research

Although SDT has been a well-established framework used to understand student motivation and achievement in various contexts, its application in the IPE context is still very limited. The lack of available instruments to measure SDT-related variables in IPE settings may be a barrier to the progress in SDT research and application in this context. Although a number of domain-general SDT measures are available (e.g., BPNS-G), these may not fully capture the nuances of IPE students’ motivation. In addition, these instruments may not provide specific information that can be used to inform IPE. Hence, in this research, we adapted and validated the BPNG-S to cater specifically for the IPE setting and to demonstrate how the instrument could be used in examining IPE outcomes.

Hence, in the current study, we addressed these issues by adapting and applying the BPNG-S to cater specifically to the IPE setting. We conducted two studies to achieve these aims. In Study 1 (Scale Validation), we contextualized the SDT framework to IPE through the adaptation and validation of the BPNS-IPE. In Study 2 (Cross-sectional Research), we demonstrated how SDT could be applied in IPE by integrating it into the program and examining the relationship between the basic psychological needs (autonomy, competence, and relatedness) and IPE outcomes. This study can contribute to IPE research and practice by making a valid instrument available to medical education researchers. Furthermore, we integrated SDT into the IPE program and used the developed instrument to examine how the satisfaction of basic psychological needs relates with IPE outcomes. By using these robust validation and correlation approaches, we hope not only to present a valid scale for measuring psychological need satisfaction in IPE but also to guide researchers on how this scale may be used to understand students’ collaboration outcomes.

## Study 1: adaptation and validation of the BPNS-IPE

The aim of Study 1 was to adapt and validate the BPNS to IPE. The BPNS-IPE is a newly adapted measure that is predicated on BPNS-G with three factors [20,21]. To develop the BPNS-IPE version, we adapted the item wordings within the context of IPE. We particularly examined the scale’s structural and external validity [37,38]. We used confirmatory factor analysis (CFA), a multivariate statistical technique for identifying structures of a set of variables where the researcher specifies the number of factors and items based on a priori model, to examine the structural validity [39]. To examine the external validity, we explored the association of the three basic psychological needs with team cohesiveness [40] through regression analysis. Team cohesiveness is defined as ‘A dynamic process that is reflected in the tendency of a team to stick together and remain united in pursuit of its goals and objectives despite difficulties and setbacks’ [41].

## Method

### Participants and procedures

Participants were 996 undergraduate healthcare students who participated in IPE in 2020 and 2021. Of these participants, 403 (40.46%, Year 4) were from Medicine, 461 (46.28%, Year 4) from Nursing, 76 (7.63%, Year 1) from Pharmacy, and 56 (5.62%, Year 5) from Speech and Hearing Sciences. The participants’ average age was 21.63 years. There were 360 (36.2%) males and 636 (63.8%) females. These students participated in the ‘Ten-day Online Asynchronous and Synchronous IPE’ as part of the required class activity.

### Measures

#### Basic psychological need satisfaction in IPE (BPNS-IPE)

The 16-item BPNS-IPE was adapted from Johnston and Finney [21] and used to estimate the students’ perceived fulfillment of the need for autonomy (3 items), competence (6 items), and relatedness (7 items). To establish the content validity for the BPNS-IPE, the content experts slightly modified wordings to adapt to the IPE context (Appendix A). For example, the item ‘*People I know tell me I am good at what I do*’ was modified to ‘*People in my IPE team tell me I am good at what I do*.’ The scale is answerable from 1 (not at all true) to 7 (very true).

#### Team cohesiveness

We used the 4-item scale of Seashore [42] to measure team cohesiveness. We used the term ‘IPE team’ to make the items specific to the IPE context (e.g., ‘*We got along with others as IPE team*.’). This scale has been validated and used in medical education studies (e.g [43]).

### Data analysis

We checked the normality of our data, examining the skewness and kurtosis [44]. Then, we performed confirmatory factor analysis (CFA) on the three-factor model of basic psychological need satisfaction (autonomy, competence, relatedness) to test its goodness of fit. We used a number of goodness-of-fit indices to examine the model fit [45]. These indices include the following: chi-square (χ^2^), ratio of chi-square values to the degrees of freedom (χ^2^/*df*); comparative fit index (CFI), normed fit index (NFI), incremental fit index (IFI), Tucker-Lewis Index (TLI), and root mean square error of approximation (RMSEA). Guidelines indicated that the chi-square statistic should be non-significant, and RMSEA should have less than .08 to indicate an acceptable fit. Additionally, GFI, NFI, IFI, TLI, and CFI values higher than .90 show an acceptable fit. The analysis was performed using AMOS 27.

We also conducted regression analysis with the three basic psychological needs as predictors and team cohesiveness as the outcome to provide additional evidence of criterion-related validity. The regression analysis was conducted using SPSS Version 27.

### Results

We report the descriptive statistics, reliability coefficients, and correlations in Table 1. SDT subscales had alpha values ranging from .71 to .85. Significant positive correlations were found between the three basic psychological needs and team cohesiveness.

**Table 1.** Correlation among study variables (n = 996).

	Autonomy	Competence	Relatedness	Team cohesiveness
Autonomy	-	.002	.33***	.38**
(2) Competence		-	.02	.22**
(3) Relatedness			-	.21**
(4) Team cohesiveness				-
Mean	6.28	2.80	5.72	4.56
*SD*	.89	.96	.97	.57
Cronbach’s alpha	.85	.71	.80	.73

*p < .05; **p < .01.

Table 2 shows the results of CFA. The three-factor a priori model of basic psychological need satisfaction obtained a good fit for our data. This indicates the validity of this model in a sample of IPE students and hence, provides support for its applicability to the IPE context. Table 3 shows the results of the regression analysis. Among the three basic psychological needs, autonomy and relatedness were found to be significant predictors of team cohesiveness.

**Table 2.** Results of confirmatory factor analysis.

Model	Chi square	*df*	*p*	CFI	NFI	IFI	TLI	RMSEA (90% CI)
16 items	322.32	101	>.001	.92	.90	.91	.91	.04
Criteria for good fit (Hu and Bentler 1995)				>.90	>.90	>.90	>.90	.08 or lower

CFI = comparative fit index, NFI = normed fit index, IFI = incremental fit index, TLI = Tucker Lewis Index, RMESEA = root mean square error of approximation.

**Table 3.** Results of regression analysis.

Variable	Team Cohesiveness		
	*B*	*t*	*Β*
Autonomy	.36	11.57	.357***
Competence	.02	.78	.023
Relatedness	.10	3.04	.094**
*F*	62.72***		
*R*^*2*^	.159		

*p < .05, **p < .01, ***p < .001.

## Study 2: application of SDT in IPE context

In Study 2, we demonstrated the applicability of the SDT framework in IPE by integrating it into IPE. Furthermore, we used the instrument adapted and validated in Study 1, the BPNS-IPE, to examine the theoretical link between psychological needs satisfaction (autonomy, competence, and relatedness) in predicting team cohesiveness in IPE. Further, this aims to provide generalizability to the earlier study of Ganotice et al. [17] on how psychological need satisfaction can predict IPE outcomes using a different set of samples.

Past studies have provided support for the important role played by teachers and peers in the satisfaction of students’ basic psychological needs. For instance, it was found that need-supportive teaching was positively associated with students’ need satisfaction and autonomous motivation, thereby increasing their learning engagement [46]. This contention was also supported by numerous studies emphasizing the role of teachers in the fulfilment of students’ basic psychological needs and its link with motivation, engagement, and achievement [47–49]. In this study, we controlled for the effects of teacher and peer effects to understand the role of psychological needs satisfaction in predicting outcomes.

Studies have demonstrated how the fulfillment of basic psychological needs contributes to positive outcomes in team contexts. In the sports domain, need satisfaction has been found to lead to increased team potency or collective beliefs in team capacity [50]. Similarly, in corporate settings, need satisfaction has been associated with team effectiveness [51]. Recently, the importance of need satisfaction in the IPE context has also started gaining recognition, as need satisfaction and autonomous motivation have been found to be linked with positive outcomes, such as interprofessional collaboration, team effectiveness, collective dedication, behavioral engagement, and goal achievement, among others [16,17].

In this study, we targeted meeting the needs for autonomy, competence, and relatedness in the IPE program by incorporating activities that promote the satisfaction of these needs. For example, to promote autonomy, we encouraged students to represent their disciplinary expertise in the tasks. For competence, we provided them with complex and challenging activities so that they could apply their clinical and collaboration skills. As for relatedness, we had activities such as ‘name your team,’ ‘e-meet your team,’ and ‘human bingo’ to increase their team cohesiveness. Based on the tenets of SDT, we propose that the satisfaction of the needs for autonomy, competence, and relatedness predicts team cohesiveness [52].

## Method

### Participants and procedures

There were 271 healthcare students from a government-subsidized University in Hong Kong who participated in this study. These pre-licensure students were from Medicine (86, Year 4), Nursing (91, Year 4), Pharmacy (38, Year 1), and Speech and Hearing Sciences (56, Year 5). Following the TBL framework, we formed these students into interprofessional teams of 5–7 members to be involved in simulated Dementia patient management [53]. We sought the approval of the Human Research Ethics Committee for Non-Clinical Faculties of the University for this study (EA1507012). We sought consent from all participants. Data collection took place at two time points: time one was after the fifth day, and time two was after the tenth day of the ten-day interprofessional education programme.

### Measures

The BPNS-IPE was adapted and validated in Study 1.

#### Behavioral engagement

This four-item scale pertains to students’ perception of how behaviorally engaged they are [54].

#### Team effectiveness

This refers to the students’ perception of their team performance [55].

#### Collective dedication

This refers to students’ perception of how dedicated their team members are [56].

#### Goal achievement

This refers to students’ perception of how IPE allowed them to achieve collaboration competencies [17].

### Data analysis

To find out whether the satisfaction of the three basic psychological needs predicts IPE outcomes, we conducted hierarchical multiple regression. In Step 1, we entered the covariates, age, gender, peer support, and teacher support to control for these variables. In Step 2, we entered the basic psychological needs, autonomy, relatedness, and competence as the independent variables. We used the same model to predict each of the IPE outcomes, namely: behavioral engagement, team effectiveness, collective dedication, and goal achievement.

### Results

We integrated SDT into the IPE program and examined how the satisfaction of the three basic psychological needs (autonomy, relatedness, and competence) predicts team cohesiveness. Bivariate correlations (Table 4) show that all three basic psychological needs are positively associated with team cohesiveness. The results of hierarchical regression analyses (Table 5) revealed that above and beyond the variance accounted for by age, gender, peer support, and teacher support, basic psychological needs predicted the IPE outcomes significantly and positively. Specifically, autonomy predicted team effectiveness; relatedness predicted behavioral engagement, team effectiveness, collective dedication, and goal achievement, while competence predicted behavioral engagement. It appeared that sense of relatedness, a variable we emphasized in IPE, became the most important predictor of all the IPE outcomes specified.

**Table 4.** Descriptive statistics and correlations for study 2(n = 271).

	Mean	SD	1	2	3	4	5	6	7	8	9	10
Autonomy	5.89	0.81	—									
(2) Relatedness	5.24	0.95	.91**	—								
(3) Competence	5.13	0.96	.88**	.93**	—							
(4) Behavioral engagement	4.13	0.59	.72**	.75**	.74**	—						
(5) Team effectiveness	4.17	0.57	.72**	.75**	.69**	.75**	—					
(6) Collective dedication	3.95	0.67	.68**	.75**	.71**	.72**	.78**	—				
(7) Goal achievement	4.04	0.73	.75**	.79**	.75**	.74**	.73**	.68**	—			
(8) Age	21.63	2.03	0.03	0.01	0.02	−0.03	−0.01	−0.01	−0.05	—		
(9) Gender	—	—	−0.03	−0.08	−.12*	−0.02	−0.02	−0.06	0.01	0.07	—	
(10) Teacher support	5.07	0.92	.33**	.35**	.35**	.35**	.28**	.31**	.31**	−.14*	0.06	—
(11) Peer support	5.09	0.98	.32**	.35**	.35**	.34**	.27**	.29**	.30**	−.12*	0.09	.81**

*p < .05, **p < .01.

**Table 5.** Results of hierarchical multiple regression for study 2 (n = 271).

*Outcomes*	Behavioral engagement			Team effectiveness			Collective dedication			Goal achievement		
*Predictors*	*Beta*	*t*	*p-value*	*Beta*	*t*	*p-value*	*Beta*	*t*	*p-value*	*Beta*	*t*	*p-value*
*Step 1*												
Age	.018	.306	.760	.045	.761	.447	.049	.836	.404	−.011	−.179	.858
Gender	−.059	−1.015	.311	−.050	−.849	.396	−.098	−1.682	.094	−.012	−.207	.836
Teacher support	.238*	2.411	.017	.196	1.938	.054	.219*	2.196	.029	.219*	2.181	.030
Peer support	.154	1.561	.120	.120	1.188	.236	.133	1.333	.184	.122	1.210	.227
*R*^*2*^	.138 10.524***			.089 6.462***			.115 8.533***			.107 7.853***		
*F*												
*Step 2*												
Age	−.044	−1.085	.279	−.009	−.223	.824	−.004	−.088	.930	−.072	−1.927	.055
Gender	.041	.996	.320	.027	.655	.513	.002	.057	.954	.086*	2.248	.025
Teacher support	.079	1.151	.251	.033	.477	.634	.065	.916	.361	.046	.727	.468
Peer support	.000	.006	.995	−.024	−.337	.736	−.020	−.286	.775	−.040	−.623	.534
Autonomy	.146	1.464	.144	.256*	2.510	.013	−.050	−.488	.626	.172	1.843	.067
Relatedness	.344**	2.721	.007	.654**	5.056	.000	.669**	5.129	.000	.565**	4.781	.000
Competence	.272*	2.371	.018	−.145	−1.238	.217	.124	1.046	.296	.083	.775	.439
*R*^*2*^		.598			.580			.573			.649	
*F*		55.181***			51.290***			49.858***			68.713***	
*ΔR*^*2*^		.460			.490			.458			.542	

*p < .05, **p < .01, ***p < .001.

### General discussion

We conducted two studies to adapt BPNS in IPE and to demonstrate how this scale can be applied to understand factors that can explain IPE outcomes. The findings revealed that BPNS-IPE is a psychometrically sound instrument adapted specifically to the IPE context. We were also able to demonstrate how SDT can be integrated into an IPE program, and provide evidence for the importance of SDT in this context, as the psychological needs (autonomy, competence, and relatedness) predicted IPE outcomes. *Theoretically*, this study provided evidence for the construct and external validity of SDT in the IPE context. Hence, SDT can be used as a framework to understand student motivation and achievement in this setting. *Practically*, we were able to demonstrate how SDT components could be integrated into an IPE program.

This study can contribute to the extant literature on IPE and SDT by adapting an instrument that measures basic psychological needs that specifically captures the nuances and uniqueness of the IPE context. The availability of this instrument can facilitate further research in this area. It can be used to gather the information that can be utilized in developing SDT-informed IPE programs. Furthermore, with the use of a newly validated BPNS-IPE, we were able to demonstrate how this scale can be used to explain sample IPE outcomes.

Study 1 extends previous findings on the validity of the BPNS-G scale which has been adapted to different language versions and in different contexts: Physical Education, Physical Exercise, Sports, Education, Romantic Relationships, Training, and Work Domain [25]. Our data involving 996 undergraduate healthcare students IPE provided support to the a priori three-factor model of BPNS as indicated by goodness-of-fit indices. The data fit using CFA was acceptable using various goodness-of-fit indices: CFI, NFI, IFI, TLI, and RMESEA. The reliability of the scale was high, with a range from .71 to .85. Further, the sense of relatedness and sense of autonomy become positive predictors of team cohesiveness, indicating between-network validity.

In Study 2, we demonstrated how the newly validated BPNS-IPE could be used to explain IPE-related outcomes. Our results indicated that sense of relatedness becomes a consistent predictor of various outcomes, including behavioral engagement, team effectiveness, collective dedication, and goal achievement in IPE, providing support to previous literature [17]. This is interesting, considering that IPE is a program designed to cultivate interpersonal competence as one of the target team competencies. Sense of autonomy predicted team effectiveness suggesting that this feeling of genuine interest or personal endorsement in IPE promotes team effectiveness [57]. We want to note that our findings illuminate the kind of motivation of Chinese healthcare students in Hong Kong.

Our results should be interpreted through the lens of important caveats. The self-report nature of data collection is not free from social desirability bias. Further, although we collected the data over two years, our data collection was only contextualized in the IPE Dementia module, which might have failed to capture students’ psychological need satisfaction across various IPE modules. In spite of these limitations, we can conceptualize new research initiatives with the use of a newly-adapted scale. We offer researchers a guide that they may find useful to pursue. *First*, the use of a person-centered methodological approach using latent profile analytic procedure is interesting to understand the profile or degree of potential combinations of psychological need satisfaction of successful IPE teams. *Second*, the role of environthe ment or facilitating conditions in meeting the psychological needs affecting outcomes is worth studying in IPE. This can be achieved through mediational analysis. *Third*, the synergy of individual and team-level analysis may be used to examine the incremental value of team-level analysis.

The ‘take home’ message from these analyses is the importance of developing an IPE context-specific scale for use by medical educators. We also demonstrated how the scale could be used to understand factors that may explain IPE outcomes. We invite other researchers to use the newly adapted scale to help in building an evidence-based IPE model that underscores the factors of achievement and engagement in IPE in medical education.

## Appendix A

The following questions concern your feelings about your IPE experience today. Please indicate how true each of the following statement is for you given your experiences. Remember that your team mates will never know how you responded to the questions. Please use the following scale in responding to the items.1 2 3 45 67 not at all true somewhat very true

**Table:**

	1	2	3	4	5	6	7
I feel like I can make a lot of inputs to deciding how my job gets done as member of healthcare team. (Autonomy)	1	2	3	4	5	6	7
(2) I really like my teammates. I interact with my healthcare team. (Relatedness)	1	2	3	4	5	6	7
(3) Often, I feel very competent in IPE. (Competence)	1	2	3	4	5	6	7
(4) People in my IPE team tell me I am good at what I do. (Competence)	1	2	3	4	5	6	7
(5) I get along with my teammates I come into contact with. (Relatedness)	1	2	3	4	5	6	7
(6) In our IPE activity, I have a lot of social contacts. (Relatedness)	1	2	3	4	5	6	7
(7) I generally feel free to express my ideas and opinions in my IPE team. (Autonomy)	1	2	3	4	5	6	7
(8) I consider my teammates to be my friends. (Relatedness)	1	2	3	4	5	6	7
(9) I have been able to learn interesting new teamwork and collaboration skills recently. (Competence)	1	2	3	4	5	6	7
(10) People in my team care about me. (Relatedness)	1	2	3	4	5	6	7
(11)Most of the time, I feel a sense of accomplishment from what I do in IPE activities. (Competence)	1	2	3	4	5	6	7
(12) In IPE activity, I do NOT get much of a chance to show how capable I am. (Competence)	1	2	3	4	5	6	7
(13) I feel like I can pretty much be myself when I’m with my team. (Autonomy)	1	2	3	4	5	6	7
(14) The teammates I interact with seem to like me much. (Relatedness)	1	2	3	4	5	6	7
(15) I often feel very capable in IPE activities. (Competence)	1	2	3	4	5	6	7
(16) My teammates from other disciplines are generally pretty friendly towards me. (Relatedness)	1	2	3	4	5	6	7
